# Citrus *PH*5-like H^+^-ATPase genes: identification and transcript analysis to investigate their possible relationship with citrate accumulation in fruits

**DOI:** 10.3389/fpls.2015.00135

**Published:** 2015-03-09

**Authors:** Cai-Yun Shi, Rui-Qin Song, Xiao-Mei Hu, Xiao Liu, Long-Fei Jin, Yong-Zhong Liu

**Affiliations:** ^1^Key Laboratory of Horticultural Plant Biology, Ministry of Education, Huazhong Agricultural UniversityWuhan, China; ^2^Key Laboratory of Horticultural Crop Biology and Genetic Improvement (Central Region), Ministry of EducationWuhan, China

**Keywords:** abscisic acid, citrus, citrate accumulation, fruit development, plasma membrane H^+^-ATPase

## Abstract

*PH*5 is a petunia gene that encodes a plasma membrane H^+^-ATPase and determines the vacuolar pH. The citrate content of fruit cell vacuoles influences citrus organoleptic qualities. Although citrus could have *PH*5-like homologs that are involved in citrate accumulation, the details are still unknown. In this study, extensive data-mining with the *PH*5 sequence and PCR amplification confirmed that there are at least eight *PH*5-like genes (*CsPH*1-8) in the citrus genome. CsPHs have a molecular mass of approximately 100 kDa, and they have high similarity to PhPH5, AtAHA10 or AtAHA2 (from 64.6 to 80.9%). They contain 13–21 exons and 12–20 introns and were evenly distributed into four subgroups of the P3A-subfamily (*CsPH*1, *CsPH*2, and *CsPH*3 in Group I, *CsPH*4 and *CsPH*5 in Group II, *CsPH*6 in Group IV, and *CsPH*7 and *CsPH*8 in Group III together with *PhPH*5). A transcript analysis showed that *CsPH*1, 3, and 4 were predominantly expressed in mature leaves, whereas *CsPH*2 and 7 were predominantly expressed in roots, *CsPH*5 and 6 were predominantly expressed in flowers, and *CsPH*8 was predominantly expressed in fruit juice sacs (JS). Moreover, the *CsPH* transcript profiles differed between orange and pummelo, as well as between high-acid and low-acid cultivars. The low-acid orange “Honganliu” exhibits low transcript levels of *CsPH*3, *CsPH*4, *CsPH*5, and *CsPH*8, whereas the acid-free pummelo (AFP) has only a low transcript level of *CsPH*8. In addition, ABA injection increased the citrate content significantly, which was accompanied by the obvious induction of *CsPH*2, 6, 7, and 8 transcript levels. Taken together, we suggest that *CsPH*8 seems likely to regulate citrate accumulation in the citrus fruit vacuole.

## Introduction

*PH*5 is a petunia gene that encodes a plasma membrane H^+^-ATPase (P-type ATPase) and has a demonstrated function in determining the vacuolar pH (Verweij et al., 2008; Faraco et al., 2014). P-type ATPases are integral membrane proteins that are generally divided into five major and evolutionarily related subfamilies, including heavy-metal ATPases (P_1B_), Ca^2+^-ATPases [endoplasmic reticulum-type Ca^2+^-ATPase and autoinhibited Ca^2+^-ATPase (P_2A_) and P_2B_], H^+^-ATPases [autoinhibited H^+^-ATPase (P_3A_), P_3B_], putative aminophospholipid ATPases (ALA, P4), and a branch with unknown specificity (P5) (Axelsen and Palmgren, 2001; Baxter et al., 2003; Pedersen et al., 2012). P-type ATPases commonly reside in the plasma membrane and act as a primary transporter for pumping protons out of the cell, thereby creating pH and electrical potential differences across the plasmalemma (Michelet and Boutry, 1995). *PH*5 belongs to the P_3A_ subfamily of P-type ATPases, and however, it resides in the vacuolar membrane (Verweij et al., 2008). Clearly, P-type ATPases are involved in many physiological functions such as the activation of secondary transport, cellular nutrient uptake, cell expansion, stress adaptation, and plant growth and development (Michelet and Boutry, 1995; Palmgren, 2001; Gaxiola et al., 2007; Duby and Boutry, 2009; Schumacher and Krebs, 2010). Moreover, they are also involved in intracellular pH regulation (Michelet and Boutry, 1995) and have a central role in vacuole acidification (Brune et al., 2002; Verweij et al., 2008; Faraco et al., 2014).

The acidity of fleshy fruit is an important component of the fruit organoleptic quality. Acidity in the citrus juice cell or other fruit species such as strawberry and pineapple is largely related to the accumulation of citrate in the cell vacuole (Etienne et al., 2013). Citrate in the citrus fruit sarcocarp is synthesized in the mitochondria via the tricarboxylic acid cycle, and it is stored in the vacuole of juice cells (Baldwin, 1993). Specifically, citrate synthase catalyzes acetyl-CoA and oxaloacetate to form citrate (citric acid) in the mitochondria (Popova and Pinheiro De Carvalho, 1998); some citrate is then transported into the cytosol when the mitochondrial aconitase activity is partially blocked (Sadka et al., 2000); in the cytosol, most citrate will be transported into the cell vacuole and be stored there, which is accompanied by a large influx of protons mediated primarily by the vacuolar H^+^-ATPase (Müller et al., 1996; Brune et al., 2002). As the fruit matures, the vacuolar citrate enters the cytosol again and is consumed through the aconitase-γ-aminobutyrate pathway (Sadka et al., 2000; Cercós et al., 2006; Degu et al., 2011) and ATP-citrate lyase pathway (Katz et al., 2007; Hu et al., 2015). Clearly, the modulation of citrate accumulation is very important for fruit quality improvement. Most studies have shown that aconitase and H^+^-ATPase play important roles in regulating citrate accumulation in the vacuole (Bogin and Wallace, 1966; Müller et al., 1996; Sadka et al., 2000; Brune et al., 2002; Cercós et al., 2006; Terol et al., 2010; Aprile et al., 2011; Degu et al., 2011). Compared with the aconitase, however, the H^+^-ATPase characteristics and functions in regulating citrate accumulation, especially at the gene level, were still lacking.

P-type ATPase is encoded by a multigene family (Baxter et al., 2003), and to date, H^+^- ATPase genes from many plants such as *Arabidopsis thaliana* (Palmgren, 2001) and *Oryza sativa* (Arango et al., 2003) have been identified as their genome sequences were published (Pedersen et al., 2012). Moreover, some of these enzymes have been suggested or shown to play a pivotal role in regulating pH homeostasis (Baxter et al., 2005; Verweij et al., 2008; Cohen et al., 2014; Faraco et al., 2014). For example, in *Arabidopsis*, the H^+^-ATPase gene *AtAHA10* exhibited a role in vacuole acidification with effects on the vacuole morphology (Baxter et al., 2005); in petunia, the mutation of P_3A_-ATPase gene *PH*5, which resides in the vacuolar membrane, resulted in a petunia with a blue flower color and high petal pH (Verweij et al., 2008). Moreover, Faraco et al. (2014) reported on a P3B-ATPase gene called *PH*1, which also resides in the vacuolar membrane, and it is required for physical interactions with *PH*5 to hyperacidify the vacuoles. In citrus, Aprile et al. (2011) also found an *AtAHA10* homolog, which was not expressed in Faris sweet lemon, but it was highly expressed in sour lemons and was suggested to have an association with citrate accumulation in lemon juice sac cells. However, the information on acid-related H^+^-ATPase genes in citrus fruits is still scarce, although three citrus genome sequences have been published (Xu et al., 2012) (www.phytozome.net).

H^+^-ATPase genes play important roles in many physiological processes, and some genes have exhibited special roles in vacuolar acidification (Baxter et al., 2005; Verweij et al., 2008; Faraco et al., 2014). Hence, we can hypothesize that there should be H^+^-ATPase genes in the citrus genome that are involved in the modulation of vacuolar acidification. In this study, eight *PH5*-like H^+^-ATPase genes were successfully identified using the *PhPH5* or *AtAHA10* sequence to query the citrus genome databases. Their transcript characteristics were investigated in the fruits of two pairs of citrus cultivars, which differ greatly in terms of citrate accumulation, to explore which gene is possibly involved in acid accumulation. Moreover, we also investigated their responses to ABA injection since the increase of ABA enhanced citrate accumulation (Liu et al., 2014; Hu et al., 2015), and some H^+^-ATPase gene expression profiles were affected by ABA (Barkla et al., 1999; Amemiya et al., 2005).

## Materials and methods

### Plant materials

“Anliu” orange (AL, *Citrus sinensis* cv. Anliu) was selected for gene organ/tissue-specific expression analysis. Samples were collected as described before (Hu et al., 2015). “Anliu” flowers (full opened, FL) and mature leaves (ML) were collected from “Anliu” trees at the inflorescence stage, fruit juice sacs (JS) were collected from “Anliu” fruits at 123 days after flowering (DAF), and fibrous roots (RT) were harvested from “Anliu” seedlings when the seedling height was over 10 cm. The seedlings were propagated as described by Zhou et al. (2014). All the samples were treated immediately with liquid nitrogen and stored at −80°C.

In addition, fruits from the AL and “Honganliu” orange (HAL, *C. sinensis* cv. Honganliu), “HB pummelo” (HBP, *C. grandis* Osbeck cv. HB pummelo) and acid-free pummelo (AFP) from a citrus germplasm orchard in Huazhong Agricultural University (Hubei province, China) were used in the present study. AL and HAL Fruits were harvested at 170 and 220 DAF. HBP and AFP Fruits were harvested at 133 and 183 DAF. Three to five healthy fruits were randomly harvested from the tree's outer crown at each time for each cultivar. Fruit JS were separated from each fruit and mixed together. They were then ground into granules in liquid nitrogen and stored at −80°C for use.

### ABA injection

A 15-year old “Owari” Satsuma mandarin (*C. unshiu* cv. Owari) tree that was grafted onto a *Poncirus trifoliata* was selected for ABA treatment. The experimental design, time, sample collection and ABA injection were the same as in previous studies (Liu et al., 2014; Hu et al., 2015). The fruits were harvested 3 days after the last injection. The fruit JS was separated, frozen in liquid nitrogen immediately, and then stored at −80°C for further use.

### Citrate determination

The citrate in the fruit JS was measured by gas-liquid chromatography (Bartolozzi et al., 1997).

### Gene isolation and sequence analysis

The sequence for *PhPH*5 (ABC59935) or *AtAHA10* (AAB32310) was used to query the three citrus genome databases [the orange genome database is from Huazhong Agricultural University (HZAU), Wuhan, China (Xu et al., 2012); another orange genome database and a clementine genome database are found in phytozome (http://phytozome.jgi.doe.gov/pz/portal.html)] using the embedded BLAST tools. The filter criteria were set with an expected *E*-value threshold of E-30, and the function annotation was the plasma membrane ATPase in the HZAU orange genome database or the KOG function annotation is the plasma membrane H^+^-transporting ATPase in phytozome. Total RNA was isolated from “Anliu” JS by following the procedure described by Liu et al. (2006). One microgram of high-quality total RNA was used for first-strand cDNA synthesis using a PrimeScript RT Reagent kit with gDNA Eraser (TaKaRa, DALIAN, China). Gene-specific primers (Table 1, Tables S2, S4) were designed by primer 3.0 (Koressaar and Remm, 2007) based on the queried genomic sequences. The open reading frame (ORF), molecular weight and isoelectric point (pI) were predicted using the EditSeq program in Lasergene software (DNASTAR, USA). Gene intron/exon structures were analyzed by the Gene Structure Display Server (GSDS, gsds.cbi.pku.edu.cn) (Guo et al., 2007). The sequence similarities of amino acid sequences were calculated using the MegAlign program in Lasergene software. The alignment of multiple sequences was conducted using the CLUSTAL X (version 1.83) program. The phylogenetic tree was constructed by MEGA4 with the neighbor-joining method (Tamura et al., 2011).

**Table 1:** **Citrus putative *PH*5-like gene sequences and their corresponding primers for quantitative real-time PCR**.

**Gene name**	**Sequence ID**	**ORF amino acid length**	**Mol. Wt (KDa)**	**pI**	**Primer name**	**Sequence (5′–3′)**		**Amplicon size (bp)**
						**Forward primer**	**Reverse primer**	
*CsPH1*	Cs5g04360.1	955	105.098	6.24	PH1	GCTCTCACAGATTTGGTGGT	CACAGCCTCCAAAACTTCCT	165
*CsPH2*	Cs6g20570.1	957	105.299	6.16	PH2	GAGGCAGTGTTGAAGGAAGC	GGTTCCACATAAACCCCAAA	190
*CsPH3*	Ciclev10013498m	936	103.237	7.68	PH3	ACGAAGCAGTTACGGAGAAC	AGTGATTCGACGTGCCCTTT	108
*CsPH4*	Cs7g07300.1	955	105.024	6.73	PH4	GTCTTCAACCACCCGAAACA	AGCTTCACCACCGATTCAAC	154
*CsPH5*	orange1.1g002208m	954	104.949	6.66	PH5	ACCCTTCATGGGCTTCAAC	GCTTCACCACTGACTCGACA	163
*CsPH6*	Cs4g01370.1	950	104.428	6.29	PH6	CAGGGTTGAAAACCAGGATG	TCCATTGCTGTCGATGTAGG	151
*CsPH7*	Ciclev10024879m	860	94.177	5.33	PH7	ACAGGCTGTCTCAGCAAGGT	GATCTTTCTCCACCCCCTTC	165
*CsPH8*	Cs1g16150.1	953	104.923	5.89	PH8	CCGTGAAGGAATTGATTTGG	CCATGACAATGGATTCCACA	190
*Actin*	XM_006464503	–	–	–	actin	CCGACCGTATGAGCAAGGAAA	TTCCTGTGGACAATGGATGGA	200

### Quantitative real-time PCR

The total RNA of all samples was isolated according to the protocol described before (Liu et al., 2006). The first-strand cDNAs were synthesized as mentioned above. Specific primers for the targeted genes and actin gene were designed with Primer 3.0 (Koressaar and Remm, 2007) and are listed in Table 1. Additionally, before quantitative Real-Time PCR (qRT-PCR), the amplification products from each primer pair of the other three cultivars were sequenced, and it was confirmed that no nucleotide difference was found among them. qRT-PCR was performed in a 10 μL reaction volume using SYBR *Premix Ex Taq* (TaKaRa, DALIAN, China) on a LightCycler 480 Real-Time System according to the manufacturer's protocol. qRT-PCR was conducted in three biological replicates. Each biological replicate was run with two technical replicates. The reactions were started with an initial incubation at 50°C for 2 min and then 95°C for 10 min, and then subjected to 40 cycles of 95°C for 15 s and 60°C for 60 s. The Livak method (Livak and Schmittigen, 2001) was employed to calculate the relative gene expression level.

### Statistical analysis

A significance test among or between samples was evaluated by Duncan's multiple range test or Student's *t*-test in the ANOVA program of SAS (SAS Institute, Cary, USA). Differences were considered significant at *P* < 0.05.

## Results

### Data mining, identification and molecular characterization of citrus PH5-like H^+^-ATPase genes

An extensive search was performed in three citrus genome databases using the *Petunia PH5* (ABC59935) or *Arabidopsis AHA10* (AAB32310) sequence. Queries with either *PhPH5* or *AtAHA10* produced the same results and showed that there were at least 11 *PH5*-like homologs in the HZAU orange database, 9 homologs in the Phytozome orange database, and 12 homologs in the Phytozome clementine database. Moreover, each gene in the HZAU orange genome database contains 1–4 theoretical transcripts (Table S1). Based on their nucleotide sequences, we could divide them into 10 groups by CLUSTAL X analysis and MEGA4 performance (Figure S1). Specific primers (Table S2) were then designed based on the consensus sequence among the genes in each group for PCR confirmation. In the end, we successfully amplified bands of expected sizes from eight groups and failed to amplify specific bands from group I and VIII (Figure S2). Because of an obvious difference in which some pairwise identities were lower than 95% that was found among the genes of some putative *PH5*-like gene groups (Table S3), we subsequently designed other specific primers (Table S4) based on the consensus sequence in the CDS (coding DNA sequence) region to screen the target gene sequence. After reduplicative PCR amplification and sequencing, we finally confirmed that the target gene sequences in groups II, III, IV, V, VI, VII, IX, and X were highly similar to those of Cs5g04360.1, Cs6g20570.1, Ciclev10013498m, Cs7g07300.1, orange1.1g002208m, Cs4g01370.1, Ciclev10024879m, and Cs1g16150.1, respectively. The identities of the amplified sequences with their corresponding genome database sequences were over 99.0%. Therefore, the corresponding genome database sequences were used in the following sequence analysis.

**Figure 1:**
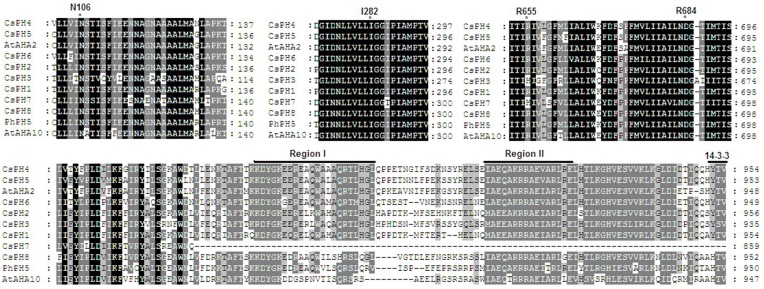
**Multiple alignment of the deduced amino acid sequence for CsPHs with the deduced amino acid residues of AtAHA2, AtAHA10, and PhPH5**. The alignment results for predicted transmembrane segments and putative autoinhibitory regions in P3A H^+^-ATPases are shown here. Asterisks mark the residues of potential importance for proton coordination and pumping based on evidence from mutagenesis and an analysis of an AtAHA2 crystal structure (Pedersen et al., 2007). The number of amino acid residues in AtAHA2 is indicated above each asterisk. R655 seems important for controlling the back flow of H^+^ at high electro-chemical gradients (Pedersen et al., 2007). The two putative autoinhibitory sequences, RegionI and RegionII, were identified by Axelsen et al. (1999), and a 14-3-3 binding site (Fuglsang et al., 1999) is shown in the C-terminal region.

The eight *PH5*-like H^+^-ATPase genes were named *CsPH*1 to 8; the corresponding genome sequence ID and basic molecular information are listed in Table 1. The peptide sequences of these eight putative *PH5*-like H^+^-ATPase genes contain 860–957 amino acids, 5.33–7.68 predicted pIs, and molecular weights from 94.177 to 105.299 kDa. They had high shared identities (Figure 1), and the identities between CsPHs were from 63.4 (between CsPH3 and CsPH7) to 90.8% (between CsPH1 and CsPH2) at the amino acid sequence level; the CsPHs also shared high similarities with PhPH5, AtAHA10, or AtAHA2, and the identities shared with PhPH5 were from 67.8 (CsPH3) to 86.6% (CsPH8), with AtAHA10 from 64.6 (CsPH3) to 80.9% (CsPH8), and for AtAHA2, they were from 70.5 (CsPH3) to 88.7% (CsPH5) (Table S5). Moreover, all CsPHs except for CsPH7, in which the C-terminal was truncated, contained two putative autoinhibitory sequences, namely Region I and Region II (Axelsen et al., 1999), and a 14-3-3 binding site (Fuglsang et al., 1999). Four residues (N_106_, I_282_, R_655_, and R_684_), which were possibly used for proton coordination and pumping (Pedersen et al., 2007), were found in all the CsPHs (Figure 1).

**Figure 2:**
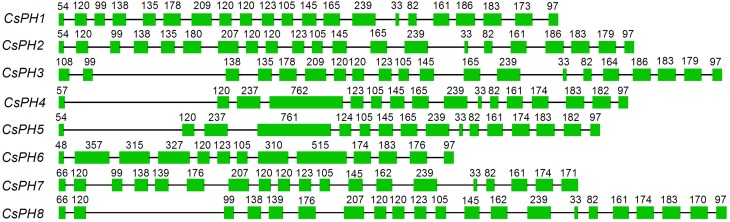
**Schematic gene structure of eight *PH5*-like H^+^-ATPases in citrus**. The boxes indicate the exon. The single line between the boxes indicates the intron. The numbers on the boxes indicate the exon length.

The full-length cDNA and gDNA sequences of the *PH5*-like H^+^-ATPase genes were downloaded from their respective genome databases. A gene structure analysis showed that the eight CsPH genes contain 13–21 exons and 12–20 introns. Specifically, *CsPH*1, 2, and 8 contain 21 exons and 20 introns; *CsPH*3 contains 20 exons and 19 introns; *CsPH*4 and *CsPH*5 contain 16 exons and 15 introns; *CsPH*6 contains 13 exons and 12 introns; and *CsPH*7 contains 19 exons and 18 introns. Moreover, most exon sizes were conserved among the CsPH genes (Figure 2).

**Figure 3:**
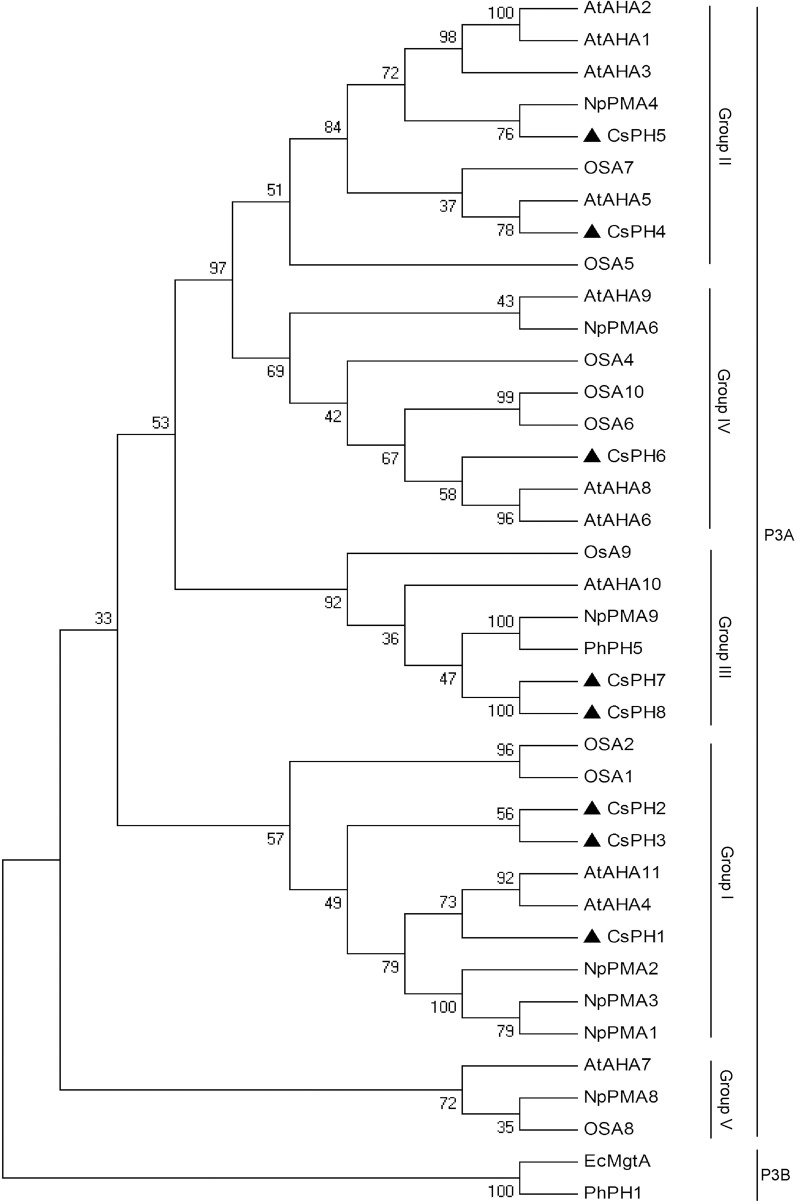
**Phylogenetic analysis of the CsPH polypeptide sequences and other P_3A_-ATPases from *Arabidopsis* (At), *Nicotiana plumbaginifolia* (Np), rice (Os), and petunia (Ph)**. The phylogenetic tree was constructed using the MEGA 4.0 program with the neighbor-joining method. The plasma membrane H^+^-ATPase gene accession numbers are listed in Table S6. The numbers at the branch points indicate bootstrap support (1000 replicates). The black triangle shows the position of eight CsPH isoforms. The EcMgtA and PhPH1 belonging to P_3B_-ATPases were used here as outgroups.

To investigate the relationship between the *CsPH* genes and other P-type ATPase genes, another 28 sequences that belonged to the P_3A_-ATPase subfamily from *A. thaliana, Nicotiana plumbaginifolia, O. sativa*, and *Petumia hybrida*, and two sequences (PhPH1 and EcMgtA) belonging to the P_3B_-ATPase subfamily from *P. hybrida* and *Escherichia coli* were used to construct a phylogenetic tree. As shown in Figure 3, all the sequences were divided into two clusters, namely P_3A_-ATPase and P_3B_-ATPase. Of these sequences, the P_3A_-ATPase could be divided into five sub-groups (Group I to V), and PhPH1 and EcMgtA were clustered together into P_3B_-ATPase. The eight CsPHs were distributed among P_3A_-ATPase clusters. Specifically, *CsPH*1, *CsPH*2, and *CsPH*3 were clustered into Group I; *CsPH*4 and *CsPH*5 were clustered into Group II; *CsPH*6 was clustered into Group IV; and *CsPH*7 and *CsPH*8 were clustered into Group III, which contained PhPH5 and AtAHA10 (Figure 3).

### Spatial expression analysis of *PH*5-like H^+^-ATPase genes

**Figure 4:**
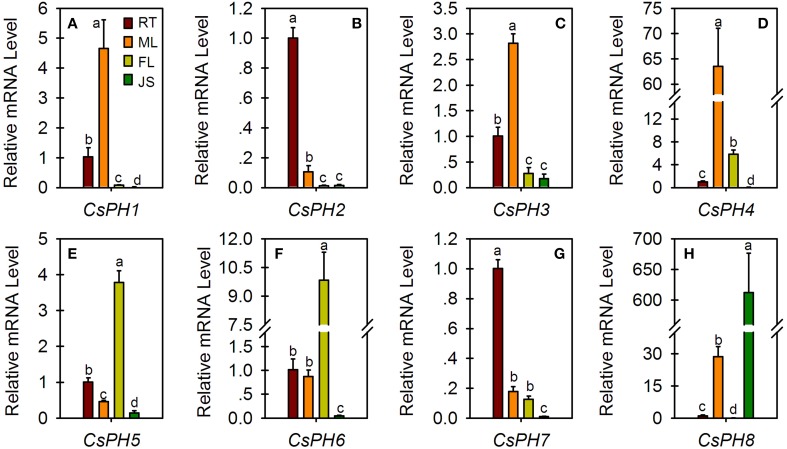
**Relative transcript levels of eight *CsPH* genes in different citrus tissues or organs**. All qRT-PCR values are the average ± Se of three replicates. Different lower-case letters on the error bar in each gene indicate significant differences at *P* < 0.05 in Duncan's multiple range test.

Expression profiles of citrus *PH5*-like H^+^-ATPase genes (*CsPH*1-8) were examined in different organs/tissues including the fruit JS of 123 DAF, FL, ML, and seedling RTs (Figure 4). *CsPH1* (Figure 4A), *CsPH3* (Figure 4C), and *CsPH4* (Figure 4D) were predominantly expressed in ML. Out of all these genes, the *CsPH1* transcript level in ML was over 4.5-, 58-, and 200- times higher than those of RT, FL, and JS, respectively (Figure 4A); the *CsPH*3 transcript level in ML was over 2.5-, 10-, and 15- times higher than those of RT, FL, and JS, respectively (Figure 4C); and the *CsPH4* transcript level in ML was over 60-, 10-, and 800- times higher than those of RT, FL, and JS, respectively (Figure 4D). On the other hand, *CsPH2* (Figure 4B) and *CsPH7* (Figure 4G) were expressed predominantly in the RT in which the *CsPH2* transcript level was over 9.5-, 76-, and 74- times higher than those of ML, FL, and JS, respectively (Figure 4B), and the *CsPH7* transcript level was over 5.5-, 7.5-, and 95- times higher than those of ML, FL, and JS, respectively (Figure 4G). In addition, *CsPH5* (Figure 4E) and *CsPH6* (Figure 4F) were predominantly expressed in FL. Unlike other citrus *PH5-like* H^+^-ATPase genes, which exhibited the lowest transcript level in JS, *CsPH8* was predominantly expressed in JS and was over 580-, 21-, and 9500- times higher than those of RT, ML, and FL, respectively (Figure 4H).

### Expression analysis of *PH*5-like H^+^-ATPase genes in the fruits from two pairs of cultivars with different citrate contents

**Figure 5:**
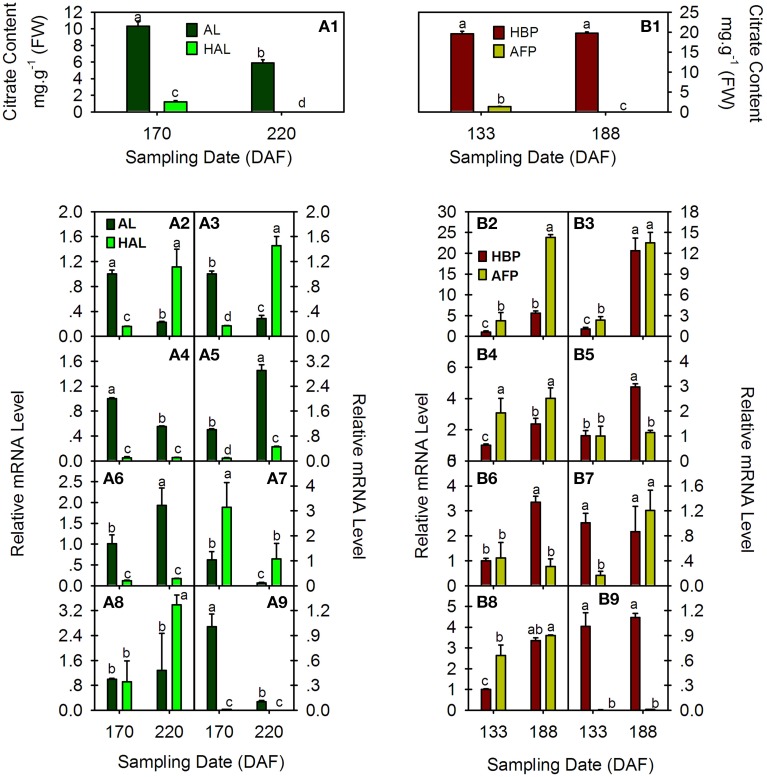
**Comparative analysis of citrate concentration (A1 and B1) and relative transcript levels of eight CsPH genes in fruit juice sacs from Anliu orange (AL), Honganliu orange (HAL), HB pummelo (HBP), and acid-free pummelo (AFP) at two developmental stages**. **A2–9** or **B2–9** refers to *CsPH*1-8. The values are the average ± Se of three replicates. Different lower-case letters on the error bar in each graph indicate a significant difference at *P* < 0.05 in Duncan's multiple range test.

The citrate content and transcript levels of citrus *PH5-like* H^+^-ATPase genes (*CsPHs*) were assayed in fruit JS at two fruit developmental stages from two pairs of cultivars, namely AL and HAL and HBP and AFP (Figure 5). HAL is a bud mutant of AL with low acidity during fruit development and ripening (Liu et al., 2007). Although their citrate contents decreased as the fruit developed, the HAL citrate content was approximately one-tenth that of AL at 170 DAF, and it was undetectable at 220 DAF (Figure 5A1). However, AFP is a low-acidity pumello and might be a pumello mutant, but its wild type is not clear. Here, we chose HBP, a relatively high-acid pumello, as a control. The HBP maintained a relatively constant acidity as the fruit developed, and the AFP citrate content decreased as the fruit developed and was undetectable at 188 DAF (Figure 5B1), similar to that of HAL.

With respect to the expression profiles of *CsPHs* in these fruits, *CsPH*1 and 2 exhibited similar expression patterns during the fruit development of AL and HAL; namely, their transcript levels decreased significantly as the AL fruit developed and increased significantly as HAL fruit developed; moreover, the *CsPH*1 or 2 transcript levels in HAL was approximately one-sixth of that in AL at 170 DAF and over three times higher than that in AL at 220 DAF (Figures 5A2,A3). Unlike AL and HAL, *CsPH*1 and 2 increased significantly in both HBP and AFP as their fruits developed; the *CsPH*1 and 2 transcript levels in AFP were significantly or slightly higher than they were in HBP at both 133 and 188 DAF (Figures 5B2,B3). *CsPH*3 and *CsPH*8 also showed similar expression patterns during fruit development in AL and HAL. As in *CsPH*1 and 2 (Figures 5A2,A3), the *CsPH*3 and 8 transcript levels clearly decreased during AL fruit development (Figures 5A4,A9); however, their transcript levels were significantly lower in HAL than those in AL and remained constant during HAL fruit development (Figures 5A4,A9), unlike *CsPH*1 and 2, which showed increasing expression patterns (Figures 5A2,A3). In HBP and AFP, *CsPH*3 and 8 showed different expression patterns from AL and HAL. The *CsPH*3 transcript level increased significantly in the high-acidity pumello (HBP) and increased slightly in the low-acidity pumello (AFP) as the fruit developed; moreover, the *CsPH*3 transcript level was obviously higher in AFP than that in HBP (Figure 5B4). The *CsPH*8 transcript level was over 10 times higher in HBP than it was in AFP at either 133 or 188 DAF; it increased slightly during HBP fruit development and remained constant during AFP fruit development (Figure 5B9). *CsPH*4 (Figures 5A5,B5) and *CsPH*5 (Figures 5A6,B6) showed similar transcript profiles during the development of high-acid cultivars including AL and HBP, for which the transcript levels increased significantly as the fruit developed. However, in the low-acid cultivars, the *CsPH*4 transcript level increased significantly in HAL (Figure 5A5) and remained constant in AFP (Figure 5B5), and the *CsPH*5 transcript level almost stayed constant in both HAL (Figure 5A6) and AFP (Figure 5B6). Moreover, the transcript levels of *CsPH*4 (Figure 5A5) and *CsPH*5 (Figure 5A6) in HAL were significantly lower than those in AL at both 170 DAF and 220 DAF, and they were significantly lower in AFP than in HBP at only 188 DAF (Figures 5B5,B6). The *CsPH*6 transcript level exhibited a distinct increase in both AL and HAL as fruit developed, although it was significantly lower in AL than in HAL at both 170 and 220 DAF (Figure 5A7). By contrast, the *CsPH*6 transcript level almost remained constant in HBP and clearly increased in AFP as the fruits developed (Figure 5B7). With respect to *CsPH*7, the transcript remained constant in AL and increased clearly in HAL as the fruits developed (Figure 5A8). In HBP and AFP, however, the *CsPH*7 transcript levels clearly increased as the fruits developed (Figure 5B8).

### Responses of the citrate content and *PH*5-like H^+^-ATPase gene expression to ABA injection

**Figure 6:**
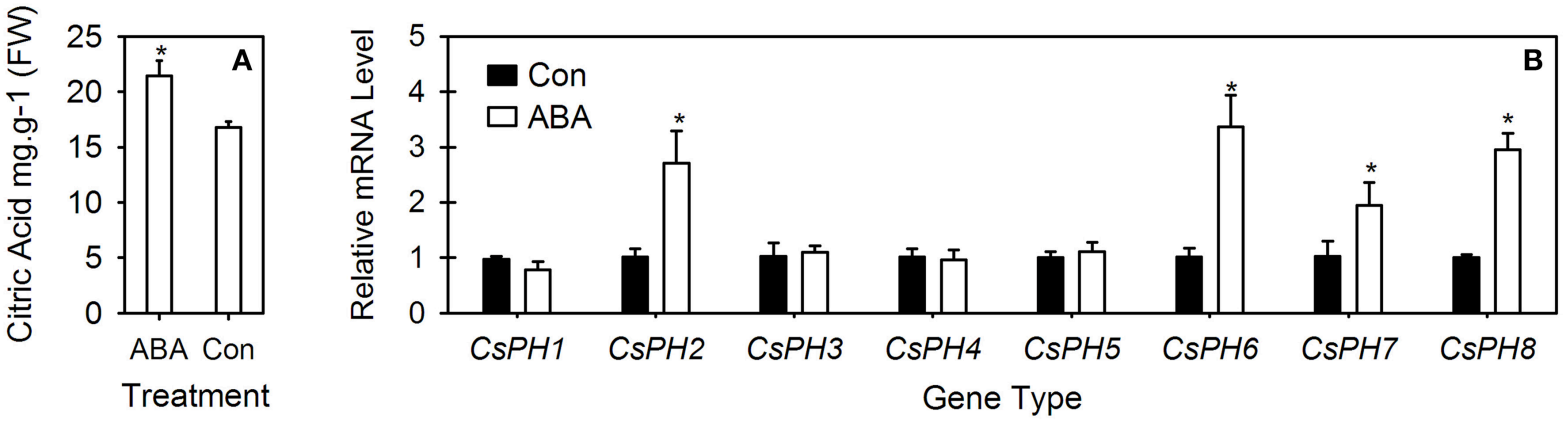
**Comparative analysis of citric acid content (A) and *CsPH1*-8 transcript levels (B) in juice sacs between ABA-injected fruits and control fruits**. The asterisk (*) on the bars indicates significant differences between the ABA treatment and control (Con) for citric acid or each gene at *P* < 0.05 based on Student's *t*-test (LSD).

The citrate content and eight *CsPH* gene transcripts were assayed in the ABA-injected fruits (Figure 6). In comparison with the control fruit in which the citrate content was 16.8 mg.g^−1^, the ABA injection significantly increased the citrate content to 21.5 mg.g^−1^ (Figure 6A). Moreover, the transcript levels of *CsPH*2, *CsPH*6, *CsPH*7, and *CsPH*8 were significantly induced by ABA injection, which were at least twice as high as their respective controls; however, the transcript levels of *CsPH*1, *CsPH*3, *CsPH*4, and *CsPH*5 showed no obvious response to ABA injection (Figure 6B).

## Discussion

Plants have at least three distinct proton pumps, that is, a P-type ATPase, a vacuolar-type proton pump [including vacuolar H^+^-pyrophosphatase (V-PPase) and vacuolar H^+^-ATPase (V-ATPase)], and an F-type ATPase. In general, the P-type ATPase pumps protons from the cytoplasm through the cell membrane, whereas V-PPase and V-ATPase acidify the intracellular compartments including the vacuole, and the F-type ATPase is evolutionarily and functionally related to V-ATPase but is confined to only the mitochondrion and chloroplast and is primarily an ATP synthase. Moreover, the P-type ATPase and V-PPase consist of a single polypeptide whereas the V- and F- ATPases are complex arrays of subunits (Perzov et al., 2001; Coker et al., 2003; Gaxiola et al., 2007; Eisenach et al., 2014).

The pH of plant endomembrane compartments is known to be regulated by V-ATPase and V-PPase, whereas the P-type ATPase controls the cytoplasm pH and energizes the plasma membrane (Gaxiola et al., 2007; Eisenach et al., 2014). However, recent research indicated that the P-type ATPase also had a role in vacuolar acidification (Baxter et al., 2005; Verweij et al., 2008; Aprile et al., 2011; Cohen et al., 2014; Faraco et al., 2014). This finding is especially true for petunia *PH5*, and Verweij et al. (2008) demonstrated that it encodes a P_3A_-ATPase proton pump, resides in the vacuolar membrane, and determines the vacuolar pH. In the present study, we searched the current three citrus genome databases using the petunia *PH5* sequence and finally identified eight *PH5*-like genes (*CsPH*1-8) from *C. sinensis* fruits (Table 1 and Figure S2). These proteins have a molecular mass of approximately 100 kDa (Table 1), which is consistent with other plant P-type ATPases, and they showed relatively high similarities to PhPH5, AtAHA2, and AtAHA10 (Table S5). Moreover, a crystal structure analysis of AtAHA2 by Pedersen et al. (2007) showed that they have the following four conserved residues: H^+^ acceptor/donor Asp684, proposed gate-keeper residue Asn106, Arg655, which was proposed to prevent H^+^ backflow, and Ile282, which is important for H^+^ transport. In addition, two clusters of autoinhibitory sequences, namely Region I and Region II (Axelsen et al., 1999), and a 14-3-3 binding site (Fuglsang et al., 1999) have been identified in the C-terminal end of P-type ATPases. Here, an alignment showed that the eight CsPHs except for CsPH7 contain the two autoinhibitory regions and a 14-3-3 binding site (Figure 1), which indicated that the eight putative P-type ATPases could have a function in the H^+^ transport in cell compartments.

The P-type ATPase is encoded by a multigene family (Pedersen et al., 2012). Extensive searches of cDNA and genomic libraries showed that there are 9, 12, and 10 P-type ATPases in *N. plumbaginifolia, A. thaliana*, and *O. sativa*, respectively (Baxter et al., 2003). All the P-type ATPase genes can be generally divided into five major evolutionarily related subfamilies (Palmgren, 2001; Arango et al., 2003; Baxter et al., 2003; Pedersen et al., 2012). Moreover, the P_3A_, though considered to be the least divergent branch of the P-Type ATPases superfamily, could be subdivided into five subfamilies (Arango et al., 2003). In this study, we found at least 11 *PH5*-like homologs in the HZAU orange database, with 9 homologs in the Phytozome orange database, and 12 homologs in the Phytozome clementine database (Table S1). However, only eight CsPHs were successfully amplified from citrus fruit (Figure S2). Previous reports indicated that not all P-type ATPase genes are expressed in all tissues (Arango et al., 2003). In fact, a spatial analysis from the present study showed that only *CsPH8* was predominantly expressed more highly in JS against RT, ML, and FL (Figure 4H). Hence, we suggest that the failure to amplify other sequences may be explained by the fact that they were not expressed in the fruits or the transcript levels are instantaneous. A comprehensive analysis of the gene structure for the successfully amplified genes subsequently showed that the numbers of exons and introns varied among the CsPHs (Figure 2). Moreover, a phylogenetic analysis showed that the eight CsPHs were distributed into four subgroups (Figure 3), which were consistent with the previous reports in other plants (Arango et al., 2003; Verweij et al., 2008), which is indicative of CsPH reliability.

The P-type ATPase has been suggested to be involved in various physiological processes (Duby and Boutry, 2009). Although typical P-type ATPases manipulate the cytoplasm pH and energize the plasma membrane, the vacuolar pH is regulated by V-ATPases together with V-PPase in plants (Gaxiola et al., 2007; Eisenach et al., 2014). Some P-type ATPases, for example *PhPH*5 (Verweij et al., 2008; Faraco et al., 2014), were unlike other P-type ATPases and were related to vacuolar acid homeostasis (Aprile et al., 2011; Cohen et al., 2014; Faraco et al., 2014). Here, we detected eight CsPH gene transcript levels in the fruit JS of high-citrate cultivars (AL and HBP) and low-citrate cultivars (HAL and AFP). The *CsPH* transcript levels differed between orange and pummelo, and between high-acid and low-acid cultivars (Figure 5). Regarding the AL and HAL pair, the low-acid HAL orange is accompanied by low transcript levels of *CsPH*3 (Figure 5A4), *CsPH*4 (Figure 5A5), *CsPH*5 (Figure 5A6), and *CsPH*8 (Figure 5A9). However, only the transcript levels of *CsPH*3 (Figure 5A4) and *CsPH*8 (Figure 5A9) decreased as the fruit ripened, which is consistent with the citrate decrease in AL fruits (Figure 5A1). As for the HBP and AFP pair, the AFP citrate content was less than one-tenth of the HBP citrate content, which was high and constant between 133 and 188 DAF (Figure 5B1). However, only the change in *CsPH*8 transcript levels were consistent with citrate content changes in AFP and HBP; the *CsPH*8 transcript level in AFP was significantly lower than that of HBP, and it remained constant as the HBP fruit ripened (Figure 5B9). In considering the relation between the *CsPH* transcript levels and the citrate content, we could suggest that the transcript decrease in *CsPH*3 and/or *CsPH*8 reduced the citrate influx into the vacuole of citrus fruits, participating in the decreased citrate accumulation as the citrus fruit ripens. Conversely, the low transcript level of *CsPH*8 could be attributed to the low citrate content in HAL and AFP because it is the only one that was predominantly expressed in JS (Figure 4H), which is promising for further study.

ABA has been implicated as a key component in abiotic stress responses, including those triggered by drought, NaCl, and low-temperature stress (Umezawa et al., 2010). The endogenous ABA level is always increased by abiotic stresses. Notably, ABA treatment can increase fruit sugar accumulation (Kobashi et al., 2001; Kempa et al., 2008) in addition to citrate accumulation (Kojima et al., 1995; Bastías et al., 2011; Hu et al., 2015). Interestingly, V-ATPase activity was also meditated by ABA (Barkla et al., 1999). Here, we found that ABA injection increased the citrate content significantly (Figure 6A) and the transcript levels of *CsPH*2, 6, 7, and 8, and it had no obvious effect on the transcript levels of *CsPH*1, 3, 4, and 5 (Figure 6B). Out of all these genes, the *CsPH*2, 7, and 8 transcript levels were increased over two times in comparison with the control, indicating that the citrate increase in the fruit JS is possibly related to the increased acid-related P-type ATPase activity, for which the latter is conducive to the influx of citrate into the vacuole. However, when combined with their spatio-temporal expression and their relation to citrate accumulation (Figures 4, 5), the transcript response analysis to ABA injection confirmed that *CsPH*8 seems likely to regulate citrate accumulation in the citrus fruit vacuole.

In conclusion, although the past acid-accumulation model in citrus fruit (Sadka et al., 2000) hypothesized that the driving force for cytosol citrate influx into cell vacuoles is mediated primarily by V-ATPase (Müller et al., 1996; Brune et al., 2002), a P-type *AtAHA10*-like gene is possibly associated with citric acid accumulation in lemon juice sac cells (Aprile et al., 2011). However, the exact information for this gene is still lacking. Because a P-type gene called the petunia *PH*5 gene has been experimentally confirmed to modulate the flower vacuole pH, we isolated eight citrus *PH5*-like genes using the *PhPH*5 sequence to query the citrus genome database. We found that only *CsPH*8 was predominately expressed in fruit JS; the low citrate content in HAL and AFP may be caused by low *CsPH*8 expression profiles. In addition, the current research provided an alternative possibility that P-type ATPases, such as *CsPH*8, may have a function in driving the citrate influx into the citrus fruit vacuole, similar to that of petunias (Verweij et al., 2008).

### Conflict of interest statement

The authors declare that the research was conducted in the absence of any commercial or financial relationships that could be construed as a potential conflict of interest.
