# Supplementary material for: Citrus PH5-like H+-ATPase genes: identification and transcript analysis to investigate their possible relationship with citrate accumulation in fruits
Source: Front Plant Sci. 2015 Mar 9;6:135. doi: 10.3389/fpls.2015.00135 (PMC4353184; doi:10.3389/fpls.2015.00135)
Supplement: Supplementary file 1 [file Table1.DOC]

Table S1 List of sequence IDs inquired from the three citrus genome databases by using the sequence of *Petunia* *PH5* (DQ334807) or *Arabidopsis AHA10* (S74033)

| No. | HZAU orange genome | Phytozome Orange Genome | Phytozome Clementine Genome |
| --- | --- | --- | --- |
| 1 | Cs1g16150.1* | orange1.1g002768m | Ciclev10024807m |
| 2 | Cs1g16160.1 | orange1.1g002208m | Ciclev10007368m |
| 3 | Cs4g01370.1 | orange1.1g002203m | Ciclev10007367m |
| 4 | Cs4g03700.1,Cs4g03700.2,  Cs4g03700.3,Cs4g03700.4 | orange1.1g044543m | Ciclev10027127m |
| 5 | Cs5g04360.1 | orange1.1g002176m | Ciclev10011000m |
| 6 | Cs5g08370.1 | orange1.1g005866m | Ciclev10018737m |
| 7 | Cs6g03420.1, Cs6g03420.2 | orange1.1g041450m | Ciclev10007374m |
| 8 | Cs6g03490.1 | orange1.1g003313m | Ciclev10024879m |
| 9 | Cs6g20570.1, Cs6g20570.2 | orange1.1g002151m | Ciclev10018727m |
| 10 | Cs7g07300.1,Cs7g07300.2 |  | Ciclev10013498m |
| 11 | Cs1g11870.1 |  | Ciclev10011010m |
| 12 |  |  | Ciclev10011040m |
| * The e-values for all the sequences are zero. | | | |
