# Supplementary material for: Citrus PH5-like H+-ATPase genes: identification and transcript analysis to investigate their possible relationship with citrate accumulation in fruits
Source: Front Plant Sci. 2015 Mar 9;6:135. doi: 10.3389/fpls.2015.00135 (PMC4353184; doi:10.3389/fpls.2015.00135)
Supplement: Supplementary file 2 [file Table2.DOC]

Table S2 Specific primers for the confirmation of each putative *PH5*-like gene group

| Group | Seqence IDs | Primer Name | Primer Sequence (5`-3`) | | Expected Amplicon Size (bp) |
| --- | --- | --- | --- | --- | --- |
|  |  |  | Forward primer | Reverse primer |  |
| Group I | orange1.1g002151m,Ciclev10018727m,Cs5g08370.1 | PH1 | ATGGAAAGTGGAGCGAAGAA | CAACGCCATTACCAAAAACC | 654 |
| Group II | orange1.1g005866m,Ciclev10018737m,Cs5g04360.1 | PH2 | GCTGCTCGTAGTGCTTCTGA | ATGTTCTTTGTGCATGTGCC | 826 |
| Group III | orange1.1g002176m,Ciclev10011000m,Cs6g20570.1，Cs6g20570.2 | PH3 | GCAGATGCTCGTCTCCTTGA | GAGGGCTGTTCGCTTATCTG | 738 |
| Group IV | Ciclev10011040m,Ciclev10013498m,Ciclev10011010m,Cs6g03490.1，Cs6g03420.1，Cs6g03420.2 | PH4 | CTGGAAGCATTCTTGGTGGT | ATTGCGTCGATATCCAGTCC | 703 |
| Group V | orange1.1g002203m,Ciclev10007367m,Cs7g07300.1，Cs7g07300.2 | PH5 | ACAGATGCTGCCAGAAGTGC | CTTCACCACCGATTCAACAT | 978 |
| Group VI | orange1.1g002208m,Ciclev10007368m,Cs4g03700.1，Cs4g03700.2，Cs4g03700.3，Cs4g03700.4 | PH6 | AGATGCTGCAAGAGGTGCTT | AAGCCCATGAAGGGTTCTTT | 829 |
| Group VII | orange1.1g041450m,Ciclev10007374m,Cs4g01370.1 | PH7 | GTACGGACAGCGATGGTCTT | GGAAGATGGCTCTGCTTGTC | 864 |
| Group VIII | orange1.1g044543m,Ciclev10027127m,Cs1g11870.1 | PH8 | CCGTATTGTGCTGGGTTTTT | TTAGCCTTGCAACTTCAGCA | 890 |
| Group IX | orange1.1g003313m,Ciclev10024879m,Cs1g16160.1 | PH9 | ACTCACTGTTGACCGGAACC | AAGGTAGCCACCAATTGAGC | 1237 |
| Group X | orange1.1g002768m,Ciclev10024807m,Cs1g16150.1 | PH10 | TCTCAGCAGGGTGCCATTAC | GATTCCAGGCTTCTCCACTT | 1606 |
