# Supplementary material for: Citrus PH5-like H+-ATPase genes: identification and transcript analysis to investigate their possible relationship with citrate accumulation in fruits
Source: Front Plant Sci. 2015 Mar 9;6:135. doi: 10.3389/fpls.2015.00135 (PMC4353184; doi:10.3389/fpls.2015.00135)
Supplement: Supplementary file 3 [file Table3.DOC]

| Table S3 Pairwise identity among genes of each putative *PH5*-like gene group | | | | | | |
| --- | --- | --- | --- | --- | --- | --- |
| Group | Sequence NO. | Sequence ID | Pairwise Identity,% | | | |
|  |  |  | 2 | 3 | 4 | 5 |
| I | 1 | orange1.1g002151m | 99.7 | 99.8 |  |  |
|  | 2 | Ciclev10018727m |  | 99.6 |  |  |
|  | 3 | Cs5g08370.1 |  |  |  |  |
| II | 1 | orange1.1g005866m | 96.4 | 96.3 |  |  |
|  | 2 | Ciclev10018737m |  | 99.7 |  |  |
|  | 3 | Cs5g04360.1 |  |  |  |  |
| III | 1 | orange1.1g002176m | 99.8 | 99.3 |  |  |
|  | 2 | Ciclev10011000m |  | 99.2 |  |  |
|  | 3 | Cs6g20570.1 |  |  |  |  |
| IV | 1 | Cs6g03490.1 | 90.3 | 94.3 | 87.6 | 89.2 |
|  | 2 | Ciclev10011010m |  | 96.1 | 94.4 | 96.9 |
|  | 3 | Ciclev10011040m |  |  | 95.3 | 99.8 |
|  | 4 | Ciclev10013498m |  |  |  | 95.2 |
|  | 5 | Cs6g03420.1 |  |  |  |  |
| V | 1 | orange1.1g002203m | 99.9 | 99.8 | 99.8 |  |
|  | 2 | Ciclev10007367m |  | 99.8 | 99.8 |  |
|  | 3 | Cs7g07300.1 |  |  | 99.9 |  |
|  | 4 | Cs7g07300.2 |  |  |  |  |
| VI | 1 | orange1.1g002208m | 99.3 | 99.7 | 98.7 | 96.2 |
|  | 2 | Ciclev10007368m |  | 99.9 | 99.5 | 99.7 |
|  | 3 | Cs4g03700.1 |  |  | 96.5 | 89.5 |
|  | 4 | Cs4g03700.3 |  |  |  | 97 |
|  | 5 | Cs4g03700.4 |  |  |  |  |
| VII | 1 | orange1.1g041450m | 99.7 | 99.9 |  |  |
|  | 2 | Ciclev10007374m |  | 99.7 |  |  |
|  | 3 | Cs4g01370.1 |  |  |  |  |
| VIII | 1 | orange1.1g044543m | 99.8 | 99.3 |  |  |
|  | 2 | Ciclev10027127m |  | 99.2 |  |  |
|  | 3 | Cs1g11870.1 |  |  |  |  |
| IX | 1 | orange1.1g003313m | 96.6 | 89.8 |  |  |
|  | 2 | Ciclev10024879m |  | 87.3 |  |  |
|  | 3 | Cs1g16160.1 |  |  |  |  |
| X | 1 | orange1.1g002768 | 99 | 98.5 |  |  |
|  | 2 | Ciclev10024807m |  | 98.7 |  |  |
|  | 3 | Cs1g16150.1 |  |  |  |  |
