# Supplementary material for: Citrus PH5-like H+-ATPase genes: identification and transcript analysis to investigate their possible relationship with citrate accumulation in fruits
Source: Front Plant Sci. 2015 Mar 9;6:135. doi: 10.3389/fpls.2015.00135 (PMC4353184; doi:10.3389/fpls.2015.00135)
Supplement: Supplementary file 4 [file Table4.DOC]

| Table S4　Special primers for sequence confirmation | | |
| --- | --- | --- |
| Group | Primer Name and Sequence | Primer Position* |
| II | SCY2:GCTCTCACAGATTTGGTGGT,SCY2R:TGCTTCAATCTCTCCCTGCT;  2-1F:TGGTCATTGCCACTGGTGTA,2-1R:AGCACTACGAGCAGCATCAG;  2-2F:GCTGCTCGTAGTGCTTCTGA,2-2R:ATGTTCTTTGTGCATGTGCC | -132,639;  640,1857;  1842,2668 |
| III | SCY3L:TAACACCACCTCACCAACCA,SCY3R:GGTGGCAATAACCACAGCTT;  3-1F：GCAGATGCTCGTCTCCTTGA,3-1R:TGCCCTTCGTATTGTTTCTG;  3-2F:TGCCTTTGTTTGATCCACCTA,3-2R:CAGACCTTTCAGCCTAACCA | -95,654;  501,1512;  1459,2835 |
| IV | 4-1F:GGGAGATTCAAGCTGTGGTC,4-1R:TCATTGCCAAGTAACCACCA;  SCY4L:CTGGAAGCATTCTTGGTGGT; SCY4R:ATTGCGTCGATATCCAGTCC | 559,2103;  2096,2786 |
| V | 5-1F:GAATCCTTTGTCGTGGGTCA,5-1R:GCTTGTTCAGGGTTAGAGTG;  SCY5L: TGACTGTTGACCGAAACCTG;SCY5R: CTCCTGCAGCCTCTTAACAA;  5-2F:ACAGATGCTGCCAGAAGTGC,5-2R:CTTCACCACCGATTCAACAT | 212,1029;  1030,1749;  1839,2817 |
| VI | 6-1F:CGAGGAAGTGTTTGAGCAGC,6-1R:GACAATGTCCGAAGCACCTC;  SCY6L:AGATGCTGCAAGAGGTGCTT; SCY6R:AAGCCCATGAAGGGTTCTTT | 68,1869;  1838,2667 |
| VII | 7-1F:GGGACTGACTACAGCGGAAGG,7-1R:TCTGCCAACATCCCTACAAT;  SCY7L:GTACGGACAGCGATGGTCTT, SCY7R:GGAAGATGGCTCTGCTTGTC;  7-2F:TGACGGAACCATTATGACTATCT,7-2R:GGTTTCAATGTCCAATCCCT | 99,1157;  1061,1942;  2055,2826; |
| IX | SCY9L:GAGGAAAGCAATGCTGAGAA,SCY9R:ATGGCAGCATCAATAGCATC  9-2F：ACTCACTGTTGACCGGAACC,9-2R：CAGTCATTCCAACCACATGC  9-3F：AGGCAGATGGCTTCACAGAT，9-3R：AAGGTAGCCACCAATTGAGC | 268,968;  849,1564;  1610,2339 |
| X | SCY10L:CCGTGAAGGAATTGATTTGG,SCY10R:GCCAATTGCCAGTGTAACAG  10-1F:TCTCAGCAGGGTGCCATTAC,10-1R:GATTCCAGGCTTCTCCACTT | 48,924;  937,2575; |
