# Supplementary material for: Citrus PH5-like H+-ATPase genes: identification and transcript analysis to investigate their possible relationship with citrate accumulation in fruits
Source: Front Plant Sci. 2015 Mar 9;6:135. doi: 10.3389/fpls.2015.00135 (PMC4353184; doi:10.3389/fpls.2015.00135)
Supplement: Supplementary file 5 [file Table5.DOC]

Table S5 Identity matrix for the amino acid sequences of NpPMA9, PhPH5, AtAHA10, and eight CsPH genes

|  | AtAHA2 | AtAHA10 | CsPH1 | CsPH2 | CsPH3 | CsPH4 | CsPH5 | CsPH6 | CsPH7 | CsPH8 |
| --- | --- | --- | --- | --- | --- | --- | --- | --- | --- | --- |
| PhPH5 | 74.9 | 79.5 | 75.8 | 75.6 | 67.8 | 76.6 | 76.6 | 73.1 | 80.9 | 86.6 |
| AtAHA2 |  | 70.2 | 80.6 | 79.1 | 70.5 | 86.2 | 88.7 | 82.0 | 71.4 | 75.2 |
| AtAHA10 |  |  | 71.2 | 70.6 | 64.6 | 71.3 | 71.0 | 69.6 | 76.8 | 80.9 |
| CsPH1 |  |  |  | 90.8 | 79.4 | 82.3 | 82.5 | 78.4 | 71.8 | 75.6 |
| CsPH2 |  |  |  |  | 82.5 | 80.4 | 81.8 | 78.1 | 71.4 | 75.3 |
| CsPH3 |  |  |  |  |  | 72.4 | 72.9 | 69.8 | 63.4 | 68.4 |
| CsPH4 |  |  |  |  |  |  | 90.5 | 82.9 | 72.2 | 76.2 |
| CsPH5 |  |  |  |  |  |  |  | 83.2 | 72.9 | 77.2 |
| CsPH6 |  |  |  |  |  |  |  |  | 69.5 | 74.3 |
| CsPH7 |  |  |  |  |  |  |  |  |  | 87.7 |
