# Supplementary material for: Citrus PH5-like H+-ATPase genes: identification and transcript analysis to investigate their possible relationship with citrate accumulation in fruits
Source: Front Plant Sci. 2015 Mar 9;6:135. doi: 10.3389/fpls.2015.00135 (PMC4353184; doi:10.3389/fpls.2015.00135)
Supplement: Supplementary file 7 [file Image1.PDF]

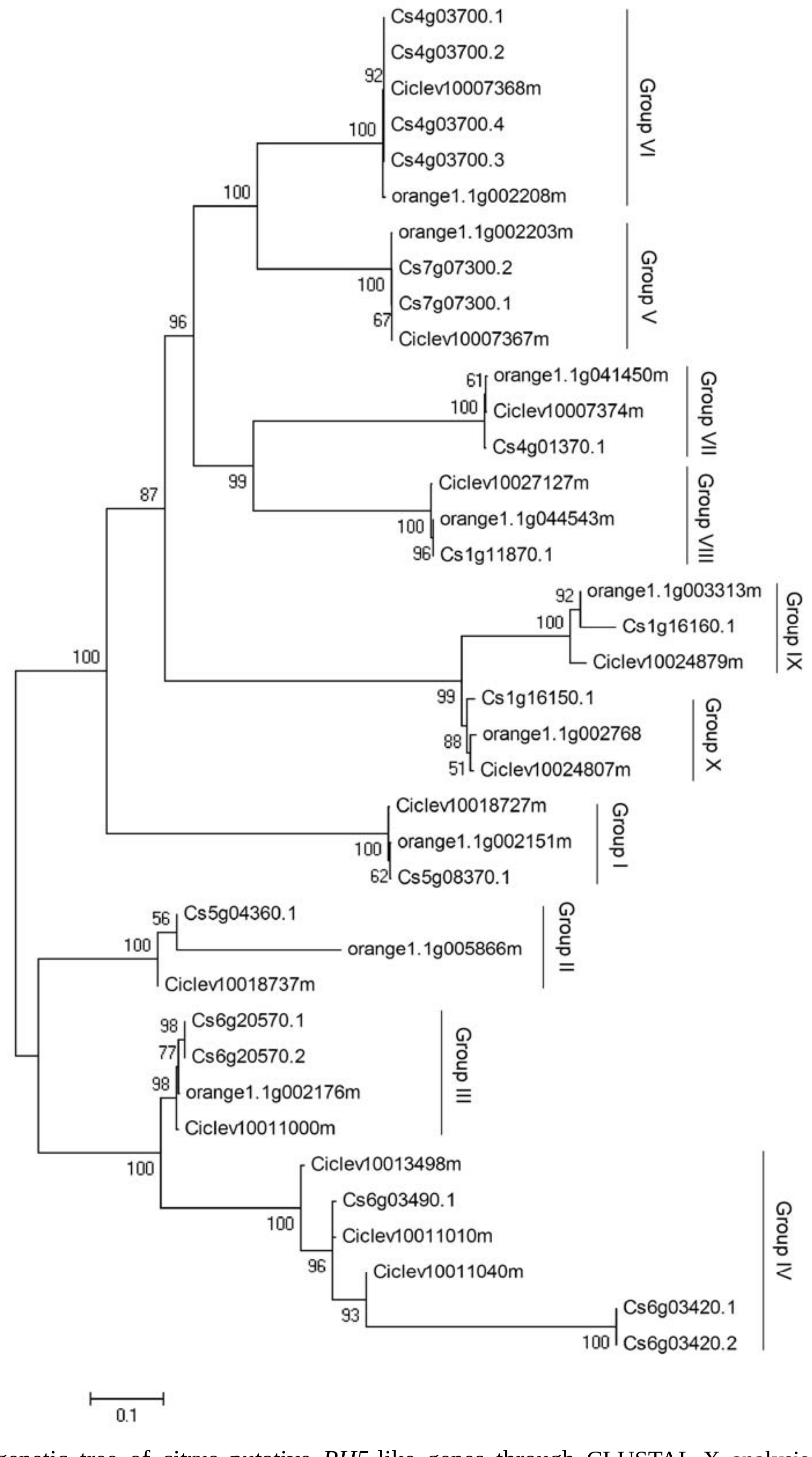

Fig.S1 Phylogenetic tree of citrus putative *PH5*-like genes through CLUSTAL X analysis and MEGA4 performance. The resulting phylogenetic tree reveals that all possible *PH5*-like genes could be divided into 10 groups.
