# Supplementary material for: Citrus PH5-like H+-ATPase genes: identification and transcript analysis to investigate their possible relationship with citrate accumulation in fruits
Source: Front Plant Sci. 2015 Mar 9;6:135. doi: 10.3389/fpls.2015.00135 (PMC4353184; doi:10.3389/fpls.2015.00135)
Supplement: Supplementary file 8 [file Image2.PDF]

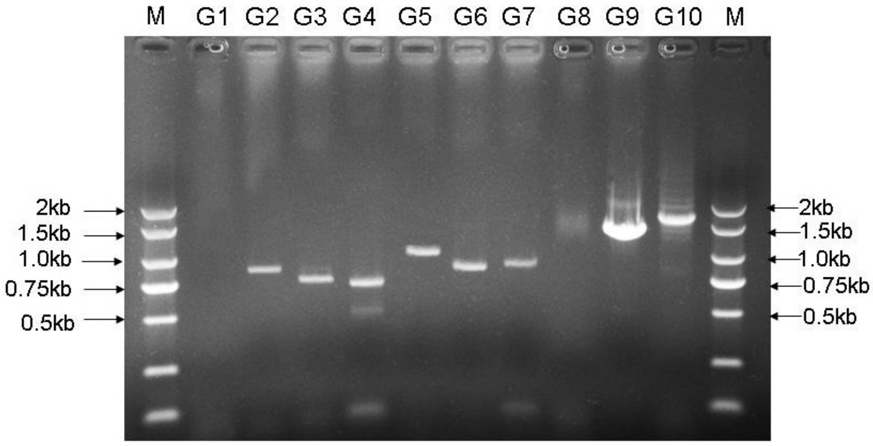

Fig. S2 Transcript confirmation of citrus *PH*5-like genes. G1 to G10 referred to Group I to X, respectively in Fig.S1. M refers to DL2000 DNA Marker(ZOMANBIO, China).
